# Supplementary material for: Interval price prediction of livestock product based on fuzzy mathematics and improved LSTM
Source: PLoS One. 2025 Feb 14;20(2):e0318823. doi: 10.1371/journal.pone.0318823 (PMC11828389; doi:10.1371/journal.pone.0318823)
Supplement: Attachment — Submitted filename: Response to Reviewers.docx [file pone.0318823.s002.docx]

Journal Requirements:

When submitting your revision, we need you to address these additional requirements.

1. Please ensure that your manuscript meets PLOS ONE's style requirements, including those for file naming. The PLOS ONE style templates can be found at https://journals.plos.org/plosone/s/file?id=wjVg/

PLOSOne_formatting_sample_main_body.pdf and https://journals.plos.or

g/plosone/s/file?id=ba62/PLOSOne_formatting_sample_title_authors_affiliations.pdf

**Response:**

Thanks very much for the following valuable comments, we have carefully revised the manuscript according to PLOS ONE's style requirements, with the detailed point-to-point responses listed as follows.

2. Please note that PLOS ONE has specific guidelines on code sharing for submissions in which author-generated code underpins the findings in the manuscript. In these cases, we expect all author-generated code to be made available without restrictions upon publication of the work. Please review our guidelines at https://journals.plos.org/plosone/s/materials-and-software-sharing#loc-sharing-code and ensure that your code is shared in a way that follows best practice and facilitates reproducibility and reuse.

**Response:**

Thanks very much for this helpful suggestion, we have shared our code in protocols.io to enhance the reproducibility of results.

DOI: dx.doi.org/10.17504/protocols.io.x54v9rj2pv3e/v1(Private link for reviewers:https://www.protocols.io/private/7AA710E9AD4811EF9F120A58A9FEAC02 to be removed before publication.)

3. Please note that funding information should not appear in any section or other areas of your manuscript. We will only publish funding information present in the Funding Statement section of the online submission form. Please remove any funding-related text from the manuscript.

**Response:**

Thanks for the careful examination. We have removed the funding-related text from the manuscript.

4. Please note that your Data Availability Statement is currently missing the repository name and/or the DOI/accession number of each dataset OR a direct link to access each database. If your manuscript is accepted for publication, you will be asked to provide these details on a very short timeline. We therefore suggest that you provide this information now, though we will not hold up the peer review process if you are unable.

**Response:**

Thanks very much for the careful examination. We have provided a direct link to access each database (https://www.ceicdata.com.cn/) and added the DOI numbers for each dataset (https://doi.org/10.3886/E211561V1).

*Based on weekly retail price data of agricultural products monitored from 500 markets across China by the Ministry of Agriculture and Rural Affairs, as sourced from CEIC Database (https://www.ceicdata.com.cn/) and illustrated in Figure 4, the prices of major livestock products in China, encompassing pork, beef, and mutton, have exhibited a notable upward trajectory since the year 2009.*

Please see the revisions in the first sentence of Section 4.1.

5. Please remove your figures from within your manuscript file, leaving only the individual TIFF/EPS image files, uploaded separately. These will be automatically included in the reviewers’ PDF.

**Response:**

Thanks for the careful examination. Accordingly, we have removed all figures from within my manuscript file.

[Note: HTML markup is below. Please do not edit.]

Reviewers' comments:

Reviewer's Responses to Questions

Comments to the Author

1. Is the manuscript technically sound, and do the data support the conclusions?

The manuscript must describe a technically sound piece of scientific research with data that supports the conclusions. Experiments must have been conducted rigorously, with appropriate controls, replication, and sample sizes. The conclusions must be drawn appropriately based on the data presented.

Reviewer #1: Yes

2. Has the statistical analysis been performed appropriately and rigorously?

Reviewer #1: Yes

3. Have the authors made all data underlying the findings in their manuscript fully available?

The PLOS Data policy requires authors to make all data underlying the findings described in their manuscript fully available without restriction, with rare exception (please refer to the Data Availability Statement in the manuscript PDF file). The data should be provided as part of the manuscript or its supporting information, or deposited to a public repository. For example, in addition to summary statistics, the data points behind means, medians and variance measures should be available. If there are restrictions on publicly sharing data—e.g. participant privacy or use of data from a third party—those must be specified.

Reviewer #1: Yes

4. Is the manuscript presented in an intelligible fashion and written in standard English?

PLOS ONE does not copyedit accepted manuscripts, so the language in submitted articles must be clear, correct, and unambiguous. Any typographical or grammatical errors should be corrected at revision, so please note any specific errors here.

Reviewer #1: Yes

5. Review Comments to the Author

Please use the space provided to explain your answers to the questions above. You may also include additional comments for the author, including concerns about dual publication, research ethics, or publication ethics. (Please upload your review as an attachment if it exceeds 20,000 characters)

Reviewer #1:

1 Please clarify the third equation for the meaning of the percentage symbol that appears.

**Response:**

Thanks for the careful examination. We have revised these minor questions in the revised paper.

*Where, x_t_ and h_t_ represent the input and output information of the hidden layer. C is the memory unit, is a candidate unit that ultimately determines which information will be added to C through the control of input gates and forget gates. W is the weight parameter matrix, and b is the bias. σ(∙) and β(∙) are the sigmoid and tanh activation functions. Attention mechanism can be introduced to improve the LSTM structure and enhance feature extraction efficiency.*

Please see the first paragraph and Formula 3 (and Formula 1 here) in Section 2.4.

$\begin{matrix} f_{t}=\sigma(W_{f}\cdot[h_{t-1},x_{t}]+b_{f}) \\ i_{t}=\sigma(W_{i}\cdot[h_{t-1},x_{t}]+b_{i}) \\ \tilde{C}=\beta(W_{C}\cdot[h_{t-1},x_{t}]+b_{C}) \\ C_{t}=f_{t}*C_{t-1}+i_{t}*\tilde{C}_{t} \\ Q_{t}=\sigma(W_{0}\cdot[h_{t-1},x_{t}]+b_{0}) \\ h_{t}=O_{t}*\tanh(C_{t}) \end{matrix}$ (1)

2 How does the accuracy of this interval prediction method compare to other interval prediction methods? It is recommended that the authors conduct an experimental comparison with other interval prediction models to validate the effectiveness of their approach.

**Response:**

According to the valuable comment, we have added comparative experiments with other interval prediction models, to validate the effectiveness of our proposed method.

*At present, the prevailing methodology for interval prediction is to construct confidence intervals by combining point prediction results with the probabilistic properties of the original data. Consequently, this comparison method is chosen as a benchmark for analysis in the study. The process commenced with the utilization of a spectrum of prevalent distribution functions, encompassing the normal, Weibull, lognormal, exponential, and gamma distributions, to model the raw data. Subsequently, interval predictions are executed at varying confidence levels (a=99%, a=95%, a=90%), leveraging the fitted distribution functions in conjunction with point prediction results. The results of these predictions are meticulously detailed in Table 5. An optimal interval prediction model aims for a lower FIAW value (i.e., a smaller prediction interval width) and a higher FICP value (i.e., the proportion of true values contained in the prediction interval). Our analysis reveals that as the confidence level decreases from 99% to 90%, the predictive certainty for these quintessential models decreases when the data aligns with their respective distributional assumptions, while the predictive accuracy increases. In terms of pork price prediction, the normal distribution has a slightly higher FIAW (e.g., 1.611), indicating a broad prediction interval and, as a result, difficulty in defining an appropriate pricing range, even while it retains some coverage proficiency at high confidence levels (e.g., FICP of 97.102 at 99%). The Weibull distribution's prediction accuracy is limited since it lacks a small FIAW, even though it achieves a FICP of 100 at a high confidence level. The lognormal distribution also struggles with a bigger FIAW at high confidence levels, resulting in an imprecise control over the price range. The exponential distribution typically exhibits a high FIAW during a range of confidence levels, with a peak FIAW of 2.024 at 99% confidence level, leading to an excessively broad spectrum of predicted prices, which is detrimental to practical applications. In both FICP and FIAW, the gamma distribution performs excellently, although it has not yet reached an optimal equilibrium state. On the other hand, the CEEMD-FE-FIG-AM-LSTM model proposed in this study has proven to have a number of benefits. With an amazing FICP value of 93.481, the model is able to approach the true value of agricultural product prices within the forecast interval, guaranteeing the prediction's trustworthiness. Meanwhile, the FIAW value is just 0.135, which is significantly less than the FIAW values of all the previously listed common distributions at different levels of confidence, signifying that the model can furnish more precise prediction intervals. Without significantly compromising the coverage of real parameters, this model provides more accurate and useful information for real-world applications by substantially decreasing the prediction range. Similar conclusions can be drawn from the beef and mutton datasets. Furthermore, it is pertinent to underscore that, despite their operational simplicity, the previously mentioned traditional interval prediction techniques do have some significant drawbacks. In particular, a common problem is the difficulty of precisely capturing the empirical distributional subtleties of prices data, as demonstrated by commodities like pork, beef, and mutton. These models' application and effectiveness in the predictive analytics field are significantly limited by their inability to properly reconcile with the inherent distributional characteristics of the data. The proposed interval prediction model, conversely, demonstrates a notable advantage in its ability to handle the intricacies of the data. It offers a more dependable and advanced prediction framework that can provide interval predictions that are both narrow and highly inclusive of the true parameters. As a result, the contributions of this study highlight the transformative potential of the suggested method within the larger framework of economic forecasting and decision-making, while also advancing the theoretical understanding of interval prediction and holding promising implications for practical applications.*

Please see the added discussion and Table 5 (and Table 1 here) in the last paragraph of Section 4.4.

**Table 1. Comparison results of the proposed model and distribution-based interval prediction methods.**

|  |  | Pork | | Beef | | Mutton | |
| --- | --- | --- | --- | --- | --- | --- | --- |
| Level | Distribution | FICP(%) | FIAW | FICP(%) | FIAW | FICP(%) | FIAW |
| a=99% | Normal | 97.102 | 1.611 | 100 | 0.823 | 100 | 0.744 |
|  | Weibull | 100 | 1.414 | 100 | 0.782 | 97.101 | 0.711 |
|  | Lognormal | 100 | 1.569 | 100 | 0.823 | 100 | 0.744 |
|  | Exponential | 100 | 2.024 | 100 | 1.669 | 100 | 1.734 |
|  | Gamma | 100 | 1.379 | 100 | 0.835 | 100 | 0.754 |
| a=95% | Normal | 92.033 | 1.226 | 55.072 | 0.626 | 50.000 | 0.566 |
|  | Weibull | 93.484 | 1.064 | 9.424 | 0.611 | 19.574 | 0.558 |
|  | Lognormal | 93.482 | 1.033 | 55.074 | 0.626 | 50.000 | 0.566 |
|  | Exponential | 100 | 1.401 | 100 | 1.155 | 100 | 1.200 |
|  | Gamma | 92.751 | 1.007 | 76.088 | 0.635 | 63.771 | 0.574 |
| a=90% | Normal | 89.862 | 1.029 | 7.245 | 0.526 | 7.246 | 0.475 |
|  | Weibull | 90.579 | 0.891 | 1.447 | 0.517 | 3.623 | 0.474 |
|  | Lognormal | 89.864 | 0.816 | 7.246 | 0.526 | 7.246 | 0.475 |
|  | Exponential | 94.926 | 1.126 | 100 | 0.928 | 100 | 0.965 |
|  | Gamma | 89.861 | 0.831 | 7.972 | 0.533 | 7.973 | 0.481 |
| **The proposed** |  | **93.481** | **0.135** | **97.832** | **0.013** | **97.828** | **0.017** |

3 It is suggested that the authors provide a more detailed description of how the prediction intervals are generated using FIG. If possible, including a flowchart to illustrate this process would greatly enhance understanding.

**Response:**

Thanks for this valuable suggestion. Accordingly, we have added a discussion on the detailed steps of the entire prediction model and visually demonstrated the generation process of the prediction interval.

*The hybrid model described in this work not only predicts the future average product value as a point, but it also predicts the future price fluctuation range as an interval. In light of the benefits of empirical modal decomposition in series smoothing, the advantages of fuzzy information granulation in the granulation interval, and the superior performance of long and short-term memory neural networks in time series data prediction, this paper selects the CEEMD decomposition model, FIG granulation model and AM-LSTM network prediction model, and also adopts the FE algorithm to recombination the components after CEEMD decomposition. Finally, we propose a combined CEEMD-FE-FIG-AM-LSTM prediction model. Fig 3 illustrates the specific steps in this procedure.*

*(1) Data composition. The initial price sequence X = {x_1_, x_2_, … , x_n_}, with a length of N, can be efficiently decomposed into serval IMF components and one residual component by CEEMD. These components capture the dynamic characteristics of the price series at different scales.*

*(2) Group allocation. The average fuzzy entropy of each IMF component is computed, serving as a criterion to assess the temporal complexity of the series. Based on this criterion, the IMF components are categorized into three groups: the first group includes components with fuzzy entropy higher than a specified threshold, the second group consists of components with fuzzy entropy lower than the threshold, and the third group encompasses the remaining components. By reducing the dimensionality of IMF components, we aim to simplify the construction complexity of the predictive model.*

*(3) Characteristics categorization. The FIG method is utilized to divide the three reconstructed modal component groups into non-overlapping, fixed-width subsequences. Each subsequence represents a time window, denoted as w_1_, w_2_, … , w_t_. Within each time window, effective information is extracted using a triangular membership function and represented by fuzzy particles G_1_, G_2_, … , Gt. These fuzzy particles form three sets of sequences: {a_1_, a_2_, … , a_t_}, {m_1_, m_2_, … , m_t_} and {b_1_, b_2_, … , b_t_}, representing low (LOW), range (R), and upper bounds (UP) respectively. These sequences reflect the average and dispersion range of price changes, where LOW and UP serve as the lower and upper bounds for interval prediction, and R represents the overall trend of price changes. To explore the influence of time intervals on the prediction system, various time windows will be selected for granulation in subsequent experiments.*

*(4) AM-LSTM prediction. AM-LSTM model is employed to train and predict for the three modal component groups separately. By incorporating the attention mechanism, the AM-LSTM model can better capture long-term dependencies in the time series data, thereby enhancing prediction accuracy.*

*(5) Forecast results superposition. For the prediction results of the three modal component groups, the final interval prediction result is obtained by linearly aggregating the LOW and UP sequences of each group, while the aggregation result of the R sequences serves as the final point prediction result. This aggregation strategy not only provides interval predictions for price changes but also offers more precise market trend analysis through point prediction results.*

Please see the revisions in the first paragraph of Section 3 and Figure 3(and Figure 1 here).

**Fig 1. Structure frame of livestock product price prediction.**

6. PLOS authors have the option to publish the peer review history of their article (what does this mean?). If published, this will include your full peer review and any attached files.

If you choose “no”, your identity will remain anonymous but your review may still be made public.

Do you want your identity to be public for this peer review? For information about this choice, including consent withdrawal, please see our Privacy Policy.

Reviewer #1: No

[NOTE: If reviewer comments were submitted as an attachment file, they will be attached to this email and accessible via the submission site. Please log into your account, locate the manuscript record, and check for the action link "View Attachments". If this link does not appear, there are no attachment files.]

While revising your submission, please upload your figure files to the Preflight Analysis and Conversion Engine (PACE) digital diagnostic tool, https://pacev2.apexcovantage.com/. PACE helps ensure that figures meet PLOS requirements. To use PACE, you must first register as a user. Registration is free. Then, login and navigate to the UPLOAD tab, where you will find detailed instructions on how to use the tool. If you encounter any issues or have any questions when using PACE, please email PLOS at <a href="mailto:figures@plos.org">figures@plos.org. Please note that Supporting Information files do not need this step.

In compliance with data protection regulations, you may request that we remove your personal registration details at any time. (Remove my information/details). Please contact the publication office if you have any questions.
