# Supplementary material for: Interval price prediction of livestock product based on fuzzy mathematics and improved LSTM
Source: PLoS One. 2025 Feb 14;20(2):e0318823. doi: 10.1371/journal.pone.0318823 (PMC11828389; doi:10.1371/journal.pone.0318823)
Supplement: Attachment — Submitted filename: Response_to_Reviewers_auresp_2.docx [file pone.0318823.s003.docx]

窗体顶端

Reviewers' comments:

Reviewer's Responses to Questions

**Comments to the Author**

1. If the authors have adequately addressed your comments raised in a previous round of review and you feel that this manuscript is now acceptable for publication, you may indicate that here to bypass the “Comments to the Author” section, enter your conflict of interest statement in the “Confidential to Editor” section, and submit your "Accept" recommendation.

Reviewer #2: (No Response)

Reviewer #3: All comments have been addressed

2. Is the manuscript technically sound, and do the data support the conclusions?

The manuscript must describe a technically sound piece of scientific research with data that supports the conclusions. Experiments must have been conducted rigorously, with appropriate controls, replication, and sample sizes. The conclusions must be drawn appropriately based on the data presented.

Reviewer #2: Yes

Reviewer #3: Yes

3. Has the statistical analysis been performed appropriately and rigorously?

Reviewer #2: Yes

Reviewer #3: Yes

4. Have the authors made all data underlying the findings in their manuscript fully available?

The [PLOS Data policy](http://www.plosone.org/static/policies.action#sharing) requires authors to make all data underlying the findings described in their manuscript fully available without restriction, with rare exception (please refer to the Data Availability Statement in the manuscript PDF file). The data should be provided as part of the manuscript or its supporting information, or deposited to a public repository. For example, in addition to summary statistics, the data points behind means, medians and variance measures should be available. If there are restrictions on publicly sharing data—e.g. participant privacy or use of data from a third party—those must be specified.

Reviewer #2: Yes

Reviewer #3: Yes

 5. Is the manuscript presented in an intelligible fashion and written in standard English?

PLOS ONE does not copyedit accepted manuscripts, so the language in submitted articles must be clear, correct, and unambiguous. Any typographical or grammatical errors should be corrected at revision, so please note any specific errors here.

Reviewer #2: No

Reviewer #3: Yes

6. Review Comments to the Author

Please use the space provided to explain your answers to the questions above. You may also include additional comments for the author, including concerns about dual publication, research ethics, or publication ethics. (Please upload your review as an attachment if it exceeds 20,000 characters)

Reviewer #2: A comprehensive livestock price prediction model with joint point and interval prediction capabilities is proposed in the work. An empirical study was conducted on the weekly price data of pork, beef, and mutton in China from 2009 to 2023, incorporating discussions on different embedding dimensions, prediction step, fuzzy granulation window sizes, decomposition techniques, and prediction algorithms. Some revisions are needed.

1. What is the number of layers of CEEMD decomposition? How does the author make sure this is optimal?

**Response:**

Thanks very much for the valuable comment. We have added the detailed steps of CEEMD decomposition in Section 2.1 and a discussion on the results in Section 4.1.3 to validate its effectiveness.

*The decomposition process of CEEMD can be expressed as:*

 *(1)*

*Where x(t) represents the original signal, Ci(t) is the i-th IMF component, and rn(t) stands for the residual component. In the CEEMD, the original signal undergoes a preprocessing step where pairs of positive and negative Gaussian white noise, denoted by u+ and u-, respectively, are added to generate positive and negative sequences.*

$\begin{matrix} x_{i}^{+}(t)=x(t)=u^{+} \\ x_{i}^{-}(t)=x(t)=u^{-} \end{matrix}$ *(2)*

*Subsequently, EMD decomposition is applied to each of these noise-added sequences, corresponding IMF components and residual components are obtained. The final result of the CEEMD decomposition is as follows:*

$\begin{matrix} IMF_{j}(t)=\frac{1}{2}\sum_{i=1}^{n} IMF_{ij}(t) \\ r(t)=\frac{1}{2}\sum_{i=1}^{n} r_{i}(t) \end{matrix}$ *(3)*

*By adopting this method, CEEMD successfully minimizes noise interference during decomposition and enhances the physical significance and interpretability of the IMFs. This study sets the chosen iteration number M to 200 and the white noise amplitude k to 0.2 based on the features of the research data.*

Please see the revisions in Section 2.1.

*Taking the pork price as an example, the price series is broken down by CEEMD to produce intrinsic mode components with various fluctuation scales. These components include six basic mode components, IMF1—IMF6 and one residual component Re. The high-frequency components (IMF1—IMF4) are thought to be the variables responsible to price unpredictability, and the low-frequency components (IMF5—IMF6) exhibit distinct periodic patterns, while the Re component reveals the original price’s long-term trend. The decomposed modal components are shown in Fig 5. Decomposing the beef price sequence in the same way yields six IMFs and one residual component, whereas the mutton price sequence also produces seven IMFs and one residual component.*

Please see the revisions in Section 4.1.3.

After CEEMD decomposition, the price data for pork, beef, and mutton are broken down into 7, 7, and 8 layers, respectively, as shown in Fig. 1. CEEMD is an advanced decomposition method that automatically determines the number of layers based on the signal's intrinsic properties and frequency components, ensuring each IMF captures a specific frequency component. The number of layers is determined adaptively without manual intervention. To optimize the CEEMD decomposition, we:

1) Preprocess the data by denoising and handling missing values.
2) Determine the optimal noise parameters through experiments (iteration

number 200 and the white noise amplitude 0.2).

3) Verify each IMF layer's compliance with the defining conditions and

check the reconstruction accuracy.

4) Compare the CEEMD method with non-decomposition approaches (as

detailed in Table 4 of the manuscript), confirming its effectiveness in separating signal components and providing reliable features for prediction.

In summary, we have ensured the optimality of the CEEMD decomposition layers, which lays a solid foundation for accurate price prediction.

**Fig 1. Decomposition results of pork, beef and mutton prices.**

2. The components are divided into three groups according to the fuzzy entropy value. The author does not make it clear here that fuzzy entropy is to be used instead of some other statistic. Why can't other statistics be used?

**Response:**

Thanks for this insightful comment. We have added a detailed explanation of the fuzzy entropy analysis performed on the decomposed sequences of pork, beef, and mutton prices in Section 4.1.3.

*Subsequently, the fuzzy entropy of these components can be calculated. For pork, the fuzzy entropy values are sequentially 0.333, 0.2891, 0.2799, 0.2346, 0.1019, 0.0401, and 0.0048; for beef, they are 0.2379, 0.2421, 0.1436, 0.0882, 0.0657, 0.0287, and 0.0153; and for mutton, they are 0.2409, 0.2372, 0.1519, 0.1174, 0.0679, 0.0307, 0.0125, and 0.0081. As the complexity decreases, the fuzzy entropy of each component gradually decreases in order. Finally, the mean fuzzy entropy of each component is computed to establish a threshold for categorizing and rearranging these components. The prices of beef and mutton have reached recombination through the same the decomposition and recombination process. Table 2 presents the recombined results. By reconstructing the original sub-modes into three new parts, the number of sub-prediction models is decreased, leading to an overall enhancement in prediction efficacy.*

Please see the revisions in Section 4.1.3.

We chose fuzzy entropy over other statistical measures to group the IMFs decomposed by CEEMD for these reasons:

1) Advantages of Fuzzy Entropy. It uses fuzzy membership functions,

making the entropy value sensitive to parameter changes. This helps accurately capture the complexity of IMFs related to price series fluctuations, providing a solid basis for grouping.

2) Robustness to noise. Fuzzy entropy reduces noise interference through mean calculations and fuzzy functions. This allows for clear identification of each IMF's true characteristics, reducing grouping errors and improving prediction accuracy.

3) Strong adaptability. Fuzzy entropy works well with data series of different lengths and characteristics. It can adapt to the varying fluctuation patterns and specificities of pork, beef, and mutton price series, aiding in the construction and optimization of AM-LSTM prediction models.

3.AM-LSTM is not a very new algorithm. Both AM and LSTM have been used in several different research efforts. And their integration is very common.

**Response:**

Thank you for your insightful comment regarding our choice of AM-LSTM for price prediction. We selected AM-LSTM for the following reasons:

1) Characteristics Match. AM-LSTM combines the attention mechanism for locating key price series information and LSTM for capturing long-term trends, making it suitable for nonlinear, non-stationary, multi-scale price data.

2) Innovative Approach. We enhanced AM-LSTM by preprocessing with CEEMD and fuzzy entropy, refining features with fuzzy information granulation, and optimizing based on price data characteristics.

3) Experimental Validation. AM-LSTM outperformed other algorithms in predicting pork, beef, and mutton prices, verifying its applicability and effectiveness.

4. Many integrated methods for RUL prediction are not discussed in the work. Such as VMD, PF and GPR are integrated to the battery RUL prediction. BLS and LSTM integrated to the battery RUL prediction. PSO, RVM and voltage for analog circuit fault prognostics, etc.

**Response:**

Thanks very much for the constructive advice. We agree that ensemble methods are important in RUL prediction. But our study focuses on forecasting pork prices, which is a complex problem due to its nonlinearity and non-stationarity. These algorithms may encounter different challenges when addressing such problems. So we have expanded our introduction to discuss these methods and justify our choice of CEEMD, FE, FIG, and AM-LSTM for livestock price series interval forecasting, and updated our references.

The fluctuation of livestock product prices exhibits high nonlinearity and instability, being intricately influenced by a multitude of complex factors such as market supply and demand, production costs, and policy regulations. This results in a high degree of complexity and uncertainty in the related data, posing a significant challenge for accurate prediction [5]. Statistical and artificial intelligence techniques are the two main approaches used to anticipate the products price [6-8]. auto regressive moving average (ARMA) Traditional statistical methods have difficulty with the highly nonlinear and unstable nature of animal commodity price data series. Data-driven models based on machine learning (ML) have emerged for agricultural product prices prediction thanks to their excellent mapping and self-learning capabilities. Many research has applied traditional Machine Learning (ML) models, including autoregressive models, back propagation neural network (BPNN), extreme learning machine (ELM) and random forest, in the field of product price prediction, encompassing livestock products, major crops, and other commodities [9-12]. Jin et al. [13] developed a Gaussian process regression (GPR) model to forecast wholesale prices of yellow corn. Zhou et al. [14] employed an optimized relevance vector machine (RVM) to accurately predict the prices of three precious metals: silver, palladium, and platinum. However, these models often encounter issues such as large prediction errors and insufficient adaptability when dealing with complex dynamic changes and large-scale datasets. Given that product prices evolve dynamically over time, there is a complex interplay between current prices and historical time series data, which profoundly reveals the causality and correlation between time series. To effectively capture these complex relationships, deep learning (DL) technologies have emerged, such as convolutional neural networks (CNN) and long short-term memory (LSTM) models, which can significantly improve the temporal dependence of time series data and capture long-term dependencies within it. Numerous studies have demonstrated the outstanding performance of the DL mode in livestock product price prediction. Nayak et al. [15] had shown that ML algorithms outperform conventional statistical methods in predicting the prices for essential crops like tomatoes, onions, and potatoes in major India markets. Gu et al. [16] proposed an innovative dual input attention LSTM model, which demonstrated high efficiency in predicting agricultural product prices. A Hidden Markov (HM) had been combined with six baseline DL models, Recurrent Neural Networks (RNN), CNN, LSTM, Gated Recurrent Units (GRU), Bidirectional LSTM and Bidirectional GRU to predict the nonlinear and nonstationary price data of agricultural commodities in [17]. Harshith et al. [18] demonstrated that time series analysis models such as Deep Neural Networks (DNN), RNN, LSTM, and GRU, are highly effective in forecasting the daily cumin prices of Unjha market, Gujarat, India. Furthermore, these deep learning techniques are also widely applied in the prediction of intrusion detection systems. These deep learning algorithms, especially LSTM [5], demonstrate robust capabilities in time series prediction, capable of finely interpreting temporal information and utilizing it to identify deep causal relationships hidden within data sequences. When dealing with datasets that are highly dependent on time, this not only enhances sensitivity to time series features, but also significantly improves the model's predictive power and accuracy by maintaining the temporal integrity of the data and capturing subtle temporal variations. However, LSTM also exhibits strong dependencies on feature selection and the configuration of model hyperparameters. Many meta-heuristic algorithms, such as particle swarm optimization (PSO) [19], crow search algorithm (COA) [5], and differential evolution (DE) [20], are often used to optimize LSTM for hyperparameter tuning, weight initialization, and other tasks. Yet, when processing large-scale datasets, these algorithms face challenges such as high computational resource consumption, complex parameter adjustment, and susceptibility to local optimization. The introduction of attention mechanisms (AM) in LSTM models [21], however, can dynamically focus on key information within the sequence, enhancing the model's attention to important features and improving the accuracy and flexibility of sequence modeling and prediction. This innovation has garnered widespread attention in the field of optimization. In order to increase the flexibility and precision of time series prediction models in a diverse data source environment, data decomposition technology has emerged as a crucial tactic. This approach reduces the difficulty of prediction tasks by using preprocessing of complicated and fluctuating time series to uncover simpler and more readable signal patterns within the data [22, 23]. Data decomposition techniques mainly include wavelet packet transform [24], empirical mode decomposition (EMD) [25-27], variational mode decomposition (VMD) [28], and other variants. Fu et al. [29] provided a robust prediction approach for pig prices based on ensemble empirical mode decomposition (EEMD) and LSTM. Li et al. [30] adopted a combination of the VMD and GRU neural network to predict the prices of beef, mutton, and pork while taking into account the effects of heterogeneous non-time series on livestock products price, such as the variety, growth cycle, longitude and latitude. Sun et al. [31] introduced a comprehensive prediction model that combined VMD, EEMD, and LSTM to forecast the average wholesale weekly price for pork, Chinese chives, shiitake mushrooms, and cauliflower. Out of all the data decomposition techniques, complementary ensemble empirical mode decomposition (CEEMD) introduces positive and negative paired noise, effectively reduces mode aliasing and endpoint effects, and exhibits excellent adaptability, hence greatly improving the stability and accuracy of decomposition. Predicting precipitation, wind speed, wave height, and other variables is another common application for CEEMD.

Please see the revisions in the second paragraph of Section 1.

Specifically, RUL prediction estimates equipment lifespan using degrading, uncertain sensor data and prefers algorithms like RVM, BLS, PF, and GPR that handle small samples, online learning, and uncertainty. Pork price prediction, influenced by seasonal and supply-demand factors, suits LSTM networks that capture long-term and nonlinear patterns. We enhanced LSTM with AM, improving over PSO-LSTM by better capturing dependencies, enhancing interpretability, automating feature extraction, reducing tuning needs, and boosting performance while reducing overfitting. In RUL, LSTM is often combined with other algorithms for better uncertainty and probabilistic predictions. For signal decomposition, CEEMD outperforms VMD, reducing mode mixing and endpoint effects, providing high-quality features for livestock price series predictions.

5. Parameter configuration, parameter adjustment scheme is very important. The author needs to give. In particular, the parameter configuration scheme of the comparison method also needs to be given.

**Response:**

Thanks for this meaningful suggestion. Accordingly, we have added a parameter specification in the revised paper.

*Three popular forecasting techniques, i.e., BPNN, ELM and traditional LSTM are introduced as significant learning paradigms. To validate the effectiveness of our proposed CEEMD-FE-FIG-AM-LSTM model, two types of models are designed for comparison: unfiltered and filtered models. Specifically, the model that makes predictions directly without any preprocessing is referred to as the unfiltered model, while the model that undergoes CEEMD, FE, or FIG processing before prediction is collectively termed the filtered model. To ensure consistency and comparability in experiments, identical specific techniques and related parameter settings are adopted with different learning paradigms. In BPNN，ReLU is chosen as the activation function, with two hidden layers configured. The first hidden layer consists of 100 neurons, and the second hidden layer contains 50 neurons. The number of iterations is set to 100, and the ADAM algorithm is utilized for optimization. In ELM, the number of hidden nodes is set to 64, and the 'sigmoid' function is adopted as the activation function. For both traditional LSTM and AM-LSTM, the number of hidden nodes is also set to 64, with the number of LSTM layers to 2, the number of training epochs to 1000, and the batch size to 32. It is worth noting that due to the inherent randomness in setting initial solutions and random parameters in these AI techniques, there may be some uncertainty in the experimental results. To comprehensively assess the robustness of the models, we measure and consider the standard deviation based on 20 runs.*

Please see the revisions in Section 4.1.2.

6. The author needs to give the calculation time of the proposed method and the calculation time of the comparison method, so as to verify the effectiveness of the proposed method.

**Response:**

Thank you for this helpful suggestion. Accordingly, we have added a discussion on the running time of the model.

*(3) From the perspective of runtime, models that have been filtered by CEEMD-FE exhibit a notable improvement in prediction accuracy despite incurring only a slight increase in computational cost compared to those without filtering. In particular, the CEEMD-FE-FIG-AM-LSTM model proposed in this paper has demonstrated a marked advantage in predictive performance across three datasets, boasting the highest FICP and the narrowest FIAW. Although this model has a relatively long runtime, it is still within an acceptable range. Specifically, in pork price prediction, the runtime of the CEEMD-FE-FIG-AM-LSTM model is 336.6 seconds, which is slightly more than the FIG-AM-LSTM (313.7 seconds) and FIG-LSTM (285.8 seconds) models that do not utilize CEEMD. However, the FICP of this model reaches as high as 93.481%, and its MAPE value is only 1.283, significantly lower than the latter two models of 6.993 and 8.166, respectively, fully demonstrating its superior prediction accuracy. In beef and mutton price prediction, the runtimes of the model are 318.7 seconds and 317.3 seconds, respectively, with minimal differences compared to models without CEEMD. But its MAPE values are lower, at 0.153 and 0.183, further validating its excellent prediction performance. In contrast, while the CEEMD-FE-FIG-ELM model has a significant advantage in runtime, with operation times of 6.7 seconds, 7.2 seconds, and 6.7 seconds for the three datasets, its prediction accuracy is relatively lower, and its FICP values are also smaller. Therefore, in practical applications, we need to strike a balance between speed and accuracy based on specific requirements. For scenarios with high real-time performance, such as real-time traffic flow prediction, the ELM model is more suitable due to its operational efficiency. Nevertheless, in situations demanding extreme prediction accuracy, such as long-term investment decisions in financial markets, electricity demand forecasting in the energy industry, or crop yield and price prediction in agriculture, the AM-LSTM model is more competitive due to its higher prediction accuracy.*

Please see the revisions in the third paragraph of Section 4.3 and Table 4 (and Table 1 here).

Table 1. Performance comparison of different prediction models.

| Dataset | Model | MAPE | RMSE | FICP | FIAW | Running time/s |
| --- | --- | --- | --- | --- | --- | --- |
| Pork | **CEEMD-FE-FIG-AM-LSTM** | **1.283** | **0.450** | **93.481** | **0.135** | **336.6** |
|  | CEEMD-FE-FIG-LSTM | 1.533 | 0.612 | 90.035 | 0.128 | 291.4 |
|  | CEEMD-FE-FIG-ELM | 2.458 | 0.771 | 84.782 | 0.112 | 6.7 |
|  | CEEMD-FE-FIG-BP | 3.781 | 1.123 | 84.233 | 0.132 | 9.1 |
|  | FIG-AM-LSTM | 6.993 | 2.899 | 75,652 | 0.114 | 313.7 |
|  | FIG-LSTM | 8.166 | 3.537 | 61.258 | 0.199 | 285.8 |
|  | FIG-ELM | 10.702 | 4.678 | 52.614 | 0.333 | 3.8 |
|  | FIG-BP | 8.372 | 3.732 | 30.429 | 0.088 | 6.2 |
| Beef | **CEEMD-FE-FIG-AM-LSTM** | **0.153** | **0.173** | **97.832** | **0.013** | **318.7** |
|  | CEEMD-FE-FIG-LSTM | 0.588 | 0.626 | 90.184 | 0.013 | 290.2 |
|  | CEEMD-FE-FIG-ELM | 1.692 | 1.544 | 64.349 | 0.012 | 7.2 |
|  | CEEMD-FE-FIG-BP | 0.392 | 0.402 | 57.829 | 0.011 | 8.1 |
|  | FIG-AM-LSTM | 1.674 | 1.574 | 76.683 | 0.009 | 314.6 |
|  | FIG-LSTM | 1.832 | 1.780 | 62.732 | 0.031 | 285.5 |
|  | FIG-ELM | 2.062 | 1.922 | 59.772 | 0.065 | 4.1 |
|  | FIG-BP | 2.164 | 1.089 | 36.124 | 0.007 | 5.0 |
| Mutton | **CEEMD-FE-FIG-AM-LSTM** | **0.183** | **0.186** | **97.828** | **0.017** | **317.3** |
|  | CEEMD-FE-FIG-LSTM | 0.188 | 0.193 | 95.284 | 0.020 | 293.2 |
|  | CEEMD-FE-FIG-ELM | 0.192 | 0.202 | 93.482 | 0.022 | 6.7 |
|  | CEEMD-FE-FIG-BP | 0.812 | 0.791 | 66.960 | 0.020 | 8.2 |
|  | FIG-AM-LSTM | 0.654 | 0.674 | 74.353 | 0.010 | 316.5 |
|  | FIG-LSTM | 0.582 | 0.612 | 62.564 | 0.012 | 298.3 |
|  | FIG-ELM | 0.492 | 0.518 | 56.957 | 0.018 | 3.8 |
|  | FIG-BP | 1.158 | 1.164 | 45.222 | 0.011 | 5.3 |

Reviewer #3: Suggest the author refer to the following literature to improve the research background and current status of the algorithm studied in this article.
[1]R. B. Kagade and N. Vijayaraj, “Intrusion detection via optimal tuned LSTM model with trust and risk level evaluation,” International Journal of Bio-Inspired Computation, vol. 23, no. 1, pp. 39–52, Jan. 2024.
[2]J. Tang, W. Gu, Z. Lei, and S. Gao, “A decode-based chaotic adaptive differential evolution for fuzzy job-shop scheduling problem,” International Journal of Bio-Inspired Computation, vol. 24, no. 4, pp. 212–222, Jan. 2024.

**Response:**

Thanks for this valuable suggestion. We've reviewed the literature you suggested and revised the introduction section on the research background and current status of the algorithm, focusing on the limitations of current time series models, LSTM's benefits, and optimization techniques. References have been updated accordingly.

The fluctuation of livestock product prices exhibits high nonlinearity and instability, being intricately influenced by a multitude of complex factors such as market supply and demand, production costs, and policy regulations. This results in a high degree of complexity and uncertainty in the related data, posing a significant challenge for accurate prediction [5]. Statistical and artificial intelligence techniques are the two main approaches used to anticipate the products price [6-8]. auto regressive moving average (ARMA) Traditional statistical methods have difficulty with the highly nonlinear and unstable nature of animal commodity price data series. Data-driven models based on machine learning (ML) have emerged for agricultural product prices prediction thanks to their excellent mapping and self-learning capabilities. Many research has applied traditional Machine Learning (ML) models, including autoregressive models, back propagation neural network (BPNN), extreme learning machine (ELM) and random forest, in the field of product price prediction, encompassing livestock products, major crops, and other commodities [9-12]. Jin et al. [13] developed a Gaussian process regression (GPR) model to forecast wholesale prices of yellow corn. Zhou et al. [14] employed an optimized relevance vector machine (RVM) to accurately predict the prices of three precious metals: silver, palladium, and platinum. However, these models often encounter issues such as large prediction errors and insufficient adaptability when dealing with complex dynamic changes and large-scale datasets. Given that product prices evolve dynamically over time, there is a complex interplay between current prices and historical time series data, which profoundly reveals the causality and correlation between time series. To effectively capture these complex relationships, deep learning (DL) technologies have emerged, such as convolutional neural networks (CNN) and long short-term memory (LSTM) models, which can significantly improve the temporal dependence of time series data and capture long-term dependencies within it. Numerous studies have demonstrated the outstanding performance of the DL mode in livestock product price prediction. Nayak et al. [15] had shown that ML algorithms outperform conventional statistical methods in predicting the prices for essential crops like tomatoes, onions, and potatoes in major India markets. Gu et al. [16] proposed an innovative dual input attention LSTM model, which demonstrated high efficiency in predicting agricultural product prices. A Hidden Markov (HM) had been combined with six baseline DL models, Recurrent Neural Networks (RNN), CNN, LSTM, Gated Recurrent Units (GRU), Bidirectional LSTM and Bidirectional GRU to predict the nonlinear and nonstationary price data of agricultural commodities in [17]. Harshith et al. [18] demonstrated that time series analysis models such as Deep Neural Networks (DNN), RNN, LSTM, and GRU, are highly effective in forecasting the daily cumin prices of Unjha market, Gujarat, India. Furthermore, these deep learning techniques are also widely applied in the prediction of intrusion detection systems. These deep learning algorithms, especially LSTM [5], demonstrate robust capabilities in time series prediction, capable of finely interpreting temporal information and utilizing it to identify deep causal relationships hidden within data sequences. When dealing with datasets that are highly dependent on time, this not only enhances sensitivity to time series features, but also significantly improves the model's predictive power and accuracy by maintaining the temporal integrity of the data and capturing subtle temporal variations. However, LSTM also exhibits strong dependencies on feature selection and the configuration of model hyperparameters. Many meta-heuristic algorithms, such as particle swarm optimization (PSO) [19], crow search algorithm (COA) [5], and differential evolution (DE) [20], are often used to optimize LSTM for hyperparameter tuning, weight initialization, and other tasks. Yet, when processing large-scale datasets, these algorithms face challenges such as high computational resource consumption, complex parameter adjustment, and susceptibility to local optimization. The introduction of attention mechanisms (AM) in LSTM models [21], however, can dynamically focus on key information within the sequence, enhancing the model's attention to important features and improving the accuracy and flexibility of sequence modeling and prediction. This innovation has garnered widespread attention in the field of optimization. In order to increase the flexibility and precision of time series prediction models in a diverse data source environment, data decomposition technology has emerged as a crucial tactic. This approach reduces the difficulty of prediction tasks by using preprocessing of complicated and fluctuating time series to uncover simpler and more readable signal patterns within the data [22, 23]. Data decomposition techniques mainly include wavelet packet transform [24], empirical mode decomposition (EMD) [25-27], variational mode decomposition (VMD) [28], and other variants. Fu et al. [29] provided a robust prediction approach for pig prices based on ensemble empirical mode decomposition (EEMD) and LSTM. Li et al. [30] adopted a combination of the VMD and GRU neural network to predict the prices of beef, mutton, and pork while taking into account the effects of heterogeneous non-time series on livestock products price, such as the variety, growth cycle, longitude and latitude. Sun et al. [31] introduced a comprehensive prediction model that combined VMD, EEMD, and LSTM to forecast the average wholesale weekly price for pork, Chinese chives, shiitake mushrooms, and cauliflower. Out of all the data decomposition techniques, complementary ensemble empirical mode decomposition (CEEMD) introduces positive and negative paired noise, effectively reduces mode aliasing and endpoint effects, and exhibits excellent adaptability, hence greatly improving the stability and accuracy of decomposition. Predicting precipitation, wind speed, wave height, and other variables is another common application for CEEMD.

Please see the revisions in the second paragraph of Section 1.

7. PLOS authors have the option to publish the peer review history of their article ([what does this mean?](https://journals.plos.org/plosone/s/editorial-and-peer-review-process#loc-peer-review-history)). If published, this will include your full peer review and any attached files.


If you choose “no”, your identity will remain anonymous but your review may still be made public.


**Do you want your identity to be public for this peer review?** For information about this choice, including consent withdrawal, please see our [Privacy Policy](https://www.plos.org/privacy-policy).

Reviewer #2: No

Reviewer #3: No

[NOTE: If reviewer comments were submitted as an attachment file, they will be attached to this email and accessible via the submission site. Please log into your account, locate the manuscript record, and check for the action link "View Attachments". If this link does not appear, there are no attachment files.]

While revising your submission, please upload your figure files to the Preflight Analysis and Conversion Engine (PACE) digital diagnostic tool, <https://pacev2.apexcovantage.com/>. PACE helps ensure that figures meet PLOS requirements. To use PACE, you must first register as a user. Registration is free. Then, login and navigate to the UPLOAD tab, where you will find detailed instructions on how to use the tool. If you encounter any issues or have any questions when using PACE, please email PLOS at [figures@plos.org](mailto:figures@plos.org). Please note that Supporting Information files do not need this step.

*In compliance with data protection regulations, you may request that we remove your personal registration details at any time.* [*(Remove my information/details)*](https://www.editorialmanager.com/pone/login.asp?a=r)*. Please contact the publication office if you have any questions.*

窗体底端
